# Penalized weighted low-rank approximation for robust recovery of recurrent copy number variations

**DOI:** 10.1186/s12859-015-0835-2

**Published:** 2015-12-10

**Authors:** Xiaoli Gao

**Affiliations:** Department of Mathematics and Statistics, University of North Carolina at Greensboro, 1400 Spring Garden St, Greensoboro, NC USA

**Keywords:** Copy number variation, Fused lasso, Low-rank approximation, Recurrent copy number variation, Penalized weighted approximation

## Abstract

**Background:**

Copy number variation (CNV) analysis has become one of the most important research areas for understanding complex disease. With increasing resolution of array-based comparative genomic hybridization (aCGH) arrays, more and more raw copy number data are collected for multiple arrays. It is natural to realize the co-existence of both recurrent and individual-specific CNVs, together with the possible data contamination during the data generation process. Therefore, there is a great need for an efficient and robust statistical model for simultaneous recovery of both recurrent and individual-specific CNVs.

**Result:**

We develop a penalized weighted low-rank approximation method (WPLA) for robust recovery of recurrent CNVs. In particular, we formulate multiple aCGH arrays into a realization of a hidden low-rank matrix with some random noises and let an additional weight matrix account for those individual-specific effects. Thus, we do not restrict the random noise to be normally distributed, or even homogeneous. We show its performance through three real datasets and twelve synthetic datasets from different types of recurrent CNV regions associated with either normal random errors or heavily contaminated errors.

**Conclusion:**

Our numerical experiments have demonstrated that the WPLA can successfully recover the recurrent CNV patterns from raw data under different scenarios. Compared with two other recent methods, it performs the best regarding its ability to simultaneously detect both recurrent and individual-specific CNVs under normal random errors. More importantly, the WPLA is the only method which can effectively recover the recurrent CNVs region when the data is heavily contaminated.

**Electronic supplementary material:**

The online version of this article (doi:10.1186/s12859-015-0835-2) contains supplementary material, which is available to authorized users.

## Background

Copy-number variations (CNVs) are changes in the number of copies of DNA in some genome regions. The size of those variations including both deletion and amplification can vary from size of 1kb to a complete chromosome arm. CNVs in genomic DNA have been considered as a major source of genomic variation [1, 2] and can be linked to the susceptibility or resistance to certain disease such as cancer, Alzheimer and Parkinson’s disease [3–5].

Three main types of technologies have been developed to detect CNVs: array comparative genomic hybridization (aCGH) arrays [6, 7], SNP genotyping arrays [8, 9] and genome re-sequencing [10–13]. Among these technologies, aCGHs have remained the most frequently used methods for CNVs identification and genotyping of personal CNVs [9, 14, 15], because of their accuracy and cost-effectiveness [16]. A typical aCGH experiment includes DNA labeling, hybridization, and scanning. During the experiment, the fluorescence signal intensities from both the test and sample DNA at given probes are measured. After some appropriate preprocessing procedures including normalization, the raw DNA copy number data from an aCGH experiment is generally in the form of log2 ratios of those intensities between test and reference DNA samples. Thus, a probe with a positive (negative) log2 ratio indicates a possible occurring of DNA amplification (deletion), while a zero value means no copy number variation is involved at this probe, that is, the copy number in the target agrees with one in the control.

However, the observed log2 intensities are often noisy surrogates of the true copy number. The detection of CNVs is to recover the underlying true copy number from the random noise at those measured positions. Many methods have been developed to analyze single-sample aCGH data, including the change-point models [17–20], smoothing methods [21–24], Haar-based wavelets [25], and Hidden Markov models [26]. Lai et al. [27] reviewed and compared the performance of some of those existing approaches.

The aforementioned methods analyze DNA CNVs data for each sample individually, which may be inefficient in the search for disease-critical genes. Recently, simultaneous analysis of multiple aCGH arrays draws considerable attention since DNA copy number regions shared by multiple samples are more likely to harbor disease-critical genes [7, 28–30]. In a multiple sample aCGH data analysis, our interest is to recover those recurrent CNV regions from the noisy data. Here a recurrent CNV region is defined as a genome region where CNVs exist for a group of samples [31].

Many of above listed individual sample CNV detection methods have been extended to recurrent CNV detection for multiple samples. Examples include hypothesis testing methods [28, 32, 33], Multiple-chain HMM [34], joint segmentation [35], and matrix factorization methods [36, 37].

Most of previous methods for recurrent CNVs detection either assume the random noise to be Gaussian distributed or ignore the co-existence of individual-specific CNVs. A multiple aCGH data is often contaminated due to either a non-normal random noise or the co-existence of outliers, i.e. heteroscedasticity, among some probes. In statistics, an outlier is an observation that is distant from other observations [38]. Some outliers may be due to intrinsic variability of the data (individual-specific effect), while others may indicate errors such as experimental error and data entry error. In a raw log2 ratio copy number data from multiple aCGH arrays, it is natural to consider the co-existence of two types of CNVs regions: recurrent CNVs regions among multiple samples and individual-specific CNVs belonging to different probes of different samples. Although the detection of recurrent CNVs is our main target, identifying some individual-specific CNVs can also help to improve our understanding of complex diseases. Moreover, the existence or mixture of individual CNVs may eventually corrupt the model fitting of recurrent CNVs, without being addressed.

Finding common or recurrent CNA regions from a noisy data remains a challenge both computationally and conceptually, not mentioning the noisy data is contaminated by both individual-specific and non-Gaussian random errors. There are much less robust methods on CNV detection from individual samples or multiple samples [39, 40]. Very recently, researchers formulated the recurrent CNV detection into a matrix decomposition problem with hidden signals being low-rank [41, 42]. For example, to address the data contamination, [41] included a individual-specific effect matrix into the model and consider the observed CNV data matrix **D** as an addition of three matrices: low-rank true recurrent CNVs matrix **X**, individual-specific effect matrix **E**, and random noise matrix ***ε***. Penalization optimization are adopted to recover **X** and **E** iteratively. In particular, a soft threshold operator [43] is adopted to update **E**. We denote this method as RPLA in this paper to differentiae it from ours. Mohammadi et al. [44] proposed another robust recurrent CNVs detection method using a Correntropy induced metric. We denote this method as CPLA in this paper. After using a Half-Quadratic technique [37, 45], CPLA is eventually reduced into a similar optimization problem as the one for RPLA. Instead of solving **E** using a soft threshold operator, CPLA updates **E** using a minimizer function *δ* during the iteration.

As explained in the last paragraph, both RPLA and CPLA introduce an individual-specific effect matrix **E** in the model needed to be estimated. She and Owen [46] has demonstrated by linear regression analysis that a Lasso penalty on the mean shift parameter cannot reduce both the masking (outliers are not detected) and swamping (normal observations are incorrectly identified as outliers) effects for the outlier detection. This justifies some limitations of RPLA, where **E** plays the same role as outliers in multiple regression in [46]. Additionally, the minimizer function *ρ* used in CPLA does not encourage the sparsity of the matrix **E**. Thus, CPLA itself does not have any ability of detecting individual-specific CNVs. This phenomenon will be further addressed in Section ‘Results and discussion’.

In this paper, we propose a novel method for robust recovery of the recurrent CNVs using a penalized weighted low-rank approximation (WPLA). Instead of using a mean shift parameter to represent each individual effect, we consider to assign a weight parameter to each probe of every sample. Thus, all the individual effects are related to a weight matrix **W**, where a weight value of 1 indicates a normal probe for a normal sample without individual-specific effect, and a weight value less than 1 indicates possible individual-specific effect occurring at this probe. We propose to shrink all individual-specific effects in the direction of the recurrent effects by penalizing the weight matrix **W**. As a result, a robust detection of recurrent CNVs is obtained by simultaneous identification of both individual-specific CNVs and recurrent ones.

Our proposed WPLA has the following two features:

- It can perform both the recurrent CNV and individual effect detection simultaneously and efficiently;
- It has strong robustness in terms of recurrent CNV detection. When the data is heavily contaminated, WPLA performs consistently better than the two aforementioned methods (CPLA and RPLA).

The rest of the paper is organized as follows. In Section ‘Methods’, we introduce our model formulation with some properties. We also provide its computation algorithm in this section. In Section ‘Results and discussion’, we demonstrate the performance of WPLA by both synthetic data analysis in multiple scenarios and two real data analysis. Finally, we conclude our paper with some discussions in Section ‘Conclusions’.

## Methods

### Formulation

Suppose we have an aCGH array data from *p* probes of *n* samples. Let *d*_*ij*_ be the observed log2 intensities at probe *j* of sample *i*. Then *d*_*ij*_ is a realization of the true hidden signal *x*_*ij*_ and random error *ε*_*ij*_,

(1) $$\begin{array}{@{}rcl@{}} d_{ij}=x_{ij}+\varepsilon_{ij}, \quad \text{for~all~}1\le i\le n, ~1\le j\le p, \end{array} $$

where *ε*_*ij*_ is assumed to have mean 0 and variance $\sigma _{\textit {ij}}^{2}=\sigma ^{2}/w_{\textit {ij}}^{2}$ for all *i* and *j*. Here 0<*w*_*ij*_≤1 is a weight parameter at probe *j* of sample *i*. A major relaxation of model (1) from existing recurrent CNVs detection methods is that we do not restrict all random errors to be homogenously distributed with the same variance. In fact, the variance can go to infinity when *w*_*ij*_ goes to 0.

Let **D**=(*d*_*ij*_), **X**=(*x*_*ij*_) and ***ε***=(*ε*_*ij*_) be three corresponding matrices from the observation data, true hidden signals, and random noises. We can write the recurrent CNV detection problem in (1) into a a multivariate regression type model,

(2) $$\begin{array}{@{}rcl@{}} \mathbf{W}\cdot(\mathbf{D}-\mathbf{X})={\boldsymbol{\varepsilon}}, \end{array} $$

where **A**·**B** represents an elementary-wise product between two matrices **A** and **B**. We consider to recover the hidden signal matrix **X** under following recurrent CNV properties:

- (P1) For each sample, the hidden log2 intensities tend to be the same at nearby probes. This property is incorporated into the model by assuming each row in the hidden signal matrix, **x**_*i*_, to be piecewise constant with only a few breakpoints.
- (P2) Most samples include recurrent CNV, and the number of unique recurrent CNV regions is small. This property is incorporated into the model by assuming the hidden signal matrix **X** to be low-rank.
- (P3) Most probes are observed with homogeneous random errors with mean 0, some of them may be contaminated and include individual-specific effects. This feature is incorporated into the model by assuming most *w*_*ij*_s to be 1, except a few of them being smaller than 1.

We propose to recover the hidden signal **X** using a penalized weighted low-rank approximation. In particular, we aim to solve an optimization problem,

(3) $$ \begin{array}{ll} {}(\widehat{\mathbf{X}}, \widehat{\mathbf{W}})=&{\mathop{\text{arg\, min}}\limits_{\mathbf{X},\mathbf{W}}}\left\{\frac{1}{2}\|\mathbf{W}\!\cdot\!(\mathbf{D}\,-\,\mathbf{X})\|_{F}^{2} + \alpha_{1}\|\mathbf{X}\|_{*}\right.\\ &+\left.\alpha_{2}\mathop{\sum}\limits_{i=1}^{n}\|\mathbf{x}_{i}\|_{\text{TV}} +\beta\|\log(\mathbf{W})\|_{1} \!\right\}\\ &\text{subject~to~} 0< \min_{i,j} w_{ij}\le \max_{i,j} w_{ij}\le 1, \end{array}  $$

where ∥**A**∥_*F*_ is the Frobenious norm. All three penalty terms in (3) are adopted to incorporate features P1–P3 for a multiple aCGH data as follows.

- The hidden signal total variation term $\alpha _{2}\|\mathbf {x}_{i}\|_{\text {TV}}=\alpha _{2}\sum _{j=2}^{p}|x_{i,j}-x_{i,j-1}|$ is to enforce a piecewise constant estimation of all **x**_*i*_ along the sequence for all 1≤*i*≤*n*, where *α*_2_>0 is a tuning parameter controlling the the number of breakpoints among all *n* sequences. The larger *α*_2_ is, the less number of breakpoints are encouraged. This term is to realize the above feature P1.
- The nuclear norm term $\alpha _{1}\|\mathbf {X}\|_{*}=\alpha _{1}\sum _{i=1}^{r} \sigma _{i}$ is adopted to realize the above feature P2 and obtain a reduced rank estimation of **X**. Here *σ*_*i*_ for 1≤*i*≤*r* are all *r* singular values of **X** and *α*_1_>0 is the tuning parameter controlling the effective rank of matrix **X**. The larger *α*_1_ is, the lower rank of **X** with stronger recurrent properties is encouraged.
- The last *ℓ*_1_ norm penalty $\beta \|\log (\mathbf {W})\|_{1}=\beta \sum _{i=1}^{n}$ $\sum _{j=1}^{p}|\log (w_{\textit {ij}})|$ is adopted to control the number of heterogeneous CNVs with individual effects. The larger *β* is, the less the individual effects is encouraged. Due to the fact that 1−*w*_*ij*_≈ log(*w*_*ij*_) when *w*_*ij*_ is close to 1, this term can be also replaced by an alternative *β*∥**1**−**W**∥_1_, where **1** is a *n*×*p* matrix with all elements being 1.

#### Robust property

The robust property of WPLA can be observed from its link with a redescending M-estimation. In particular, we can associate the WPLA approximation of **X** in (3) with a penalized *redescending* M-estimation approximation

(4) $$ {\small{\begin{aligned} {}\widehat{\mathbf{X}}_{M}\,=\,{\mathop{\text{arg\, min}}_{\mathbf{X}}}\!\left\{\!\sum_{i=1}^{n}\sum_{j=1}^{p} \rho_{\beta}(d_{ij}\,-\,x_{ij}) \,+\, \alpha_{1}\|\mathbf{X}\|_{*}\,+\,\alpha_{2}\sum_{i=1}^{n}\|\mathbf{x}_{i}\|_{\text{TV}} \!\right\}\!, \end{aligned}}}  $$

where

(5) $$\begin{array}{@{}rcl@{}} \rho_{\beta}(t)=\left\{\begin{array}{ll} \beta \log(t^{2}/\beta)+\beta & \text{if~} |t|>\sqrt{\beta}, \\ t^{2} & \text{if~} |t|\le\sqrt{\beta}. \end{array}\right. \end{array} $$

This *ρ*_*β*_(*t*) function in (5) produces strong robust property since $\frac {d \rho _{\beta }(\cdot)}{dt}$ is approaching 0 when *t*→*∞*. An additional pdf file shows more details (see Section 1 in Additional file 1).

#### Adaptive WPLA

Considering the better performance of adaptive Lasso over Lasso [47], we also propose an adaptive WPLA by minimizing,

(6) $$ {\small{\begin{array}{ll} {}\frac{1}{2}\|\mathbf{W}\cdot(\mathbf{D}-\mathbf{X})\|_{F}^{2} &+ \alpha_{1}\|\mathbf{X}\|_{*}+\alpha_{2}\sum_{i=1}^{n}\|\mathbf{x}_{i}\|_{\text{TV}}\\ &+\sum_{i=1}^{n}\sum_{j=1}^{p} \beta_{ij}|\log(w_{ij})| \\ &\text{subject~to~} 0< \min_{i,j} w_{ij}\le \max_{i,j} w_{ij}\le 1, \end{array}}}  $$

where $\beta _{\textit {ij}}=\beta /\sqrt {|\log (w^{(0)}_{\textit {ij}})|}$ and $w^{(0)}_{\textit {ij}}$ is an initial value of *w*_*ij*_. In implementation, we can obtain $w^{(0)}_{\textit {ij}}$s from $x^{(0)}_{\textit {ij}}$s using (8) to be presented in Section ‘Algorithm’, where $x^{(0)}_{\textit {ij}}$ is an initial estimate of *x*_*ij*_. For example, we can obtain $x^{(0)}_{\textit {ij}}$s from either RPLA or CPLA. If 0 occurs at the denominator, we replace it by 0.001 by convention. In the next section, we will present more details on choice of initial values following the computation algorithm for (6).

### Algorithm

Once **W** is fixed, solving **X** is a convex optimization problem. Due to the co-existence of both nuclear norm and total variation, instead of solving the optimization problem directly, we adopt the Alternating Direction Method of Multipliers (ADMM, [43]) in our algorithm. ADMM was also used in both RPLA and CPLA. We now divide the optimization problem in (3) in separate steps. Some more details on mathematical computations can be found in Additional file 1 (Section 3).

First, we rewrite the penalized objective function in model (3) as

(7) $$ {\small{\begin{array}{ll} {}L(\mathbf{X},\mathbf{W}, \mathbf{Z}, \mathbf{ Y})&=\frac{1}{2}\|\mathbf{W}\cdot(\mathbf{D}\,-\,\mathbf{X})\|_{F}^{2} + \alpha_{1}\|\mathbf{X}\|_{*}\,+\,\alpha_{2}\sum_{i=1}^{n}\|\mathbf{z}_{i}\|_{\text{TV}}\\ &\quad +\beta\|\log(\mathbf{W})\|_{1} +\!<\!\mathbf{Y},\mathbf{X}-\mathbf{Z}> +\frac{\rho}{2}\|\mathbf{X}-\mathbf{Z}\|_{F}^{2}, \end{array}}}  $$

where **Y** is the dual variable matrix, **Z** is an auxiliary variable matrix, and <**Y**,**X**−**Z**>=Tr(**Y** ^′^(**X**−**Z**)) denotes the inner product between **Y** and **X**−**Z**. Here *ρ* is a tuning parameter controlling the convergence of the algorithm (*ρ* is adaptively tuned during the iteration; one can refer Eq. (10) in Section 3.4.1 of [43] for more details).

If $\widetilde {\mathbf {X}}$ is obtained, the optimization function for solving **W** (7) becomes

$$ L(\widetilde{\mathbf{X}},\mathbf{W})\,=\, \frac{1}{2}\sum_{i=1}^{n}\sum_{j=1}^{p} w_{ij}^{2} (d_{ij}-\widetilde x_{ij})^{2} +\beta \sum_{i=1}^{n}\sum_{j=1}^{p}|\log(w_{ij})|. $$

Thus we can update *w*_*ij*_ from

(8) $$\begin{array}{@{}rcl@{}} w_{ij}=\left\{ \begin{array} {ll} \frac{\sqrt{\beta}}{|d_{ij}-\widetilde x_{ij}|} & \text{if~} |d_{ij}-\widetilde x_{ij}|>\sqrt{\beta}\\ 1 & \text{Otherwise.} \end{array} \right. \end{array} $$

If $(\widetilde {\mathbf {W}}, \widetilde {\mathbf {Z}}, \widetilde {\mathbf {Y}})$ is obtained, solving **X** from (7) becomes

(9) $$ {\small{\begin{array} {ll} {}{\mathop{\text{arg min}}\limits_{\mathbf{X}}}\{L(\mathbf{X}, \widetilde{\mathbf{W}}, \widetilde{\mathbf{Z}}, \widetilde{\mathbf{Y}})\} \\ ={\mathop{\text{arg min}}\limits_{\mathbf{X}}}\left\{\frac{1}{2}\| \widetilde{\mathbf{W}}\cdot(\mathbf{D}-\mathbf{X})\|_{F}^{2}+\alpha_{1}\|\mathbf{X}\|_{*}+<\widetilde{\mathbf{Y}},\mathbf{X}- \widetilde{\mathbf{Z}}\!>\right.\\ \quad+\left.(\rho/2)\|\mathbf{X}\,-\, \widetilde{\mathbf{Z}}\|_{F}^{2}\vphantom{\frac{1}{2}}\right\}\\ ={\mathop{\text{arg min}}\limits_{\mathbf{X}}}\left\{\mathop{\sum}\limits_{i=1}^{n}\mathop{\sum}\limits_{j=1}^{p} (\widetilde w_{ij}^{2}+\rho)\left[x_{ij}\,-\,\frac{\widetilde w_{ij}^{2}d_{ij} +\rho \widetilde z_{ij}-\widetilde y_{ij}}{\widetilde w_{ij}^{2}+\rho}\right]^{2}\right.\\ \quad +\left.2\alpha_{1}\|{\mathbf X}\|_{*}{\vphantom{\mathop{\sum}\limits_{i=1}^{n}\mathop{\sum}\limits_{j=1}^{p}}}\right\} \end{array}}}  $$

An additional pdf file shows more detailed computation (see Section 3 in Additional file 1).

It turns out that updating **X** in (9) becomes a matrix completion problem in a trace regression problem [48]. Therefore, the solution of **X** can be expressed explicitly. Denote $a_{\textit {ij}}=(\rho +\widetilde {w_{\textit {ij}}}^{2})^{1/2}$ and $b_{\textit {ij}}=a_{\textit {ij}}^{-2}(\widetilde {w_{\textit {ij}}}^{2} d_{\textit {ij}}+\rho \widetilde {z_{\textit {ij}}}-\widetilde {y_{\textit {ij}}})$. Let ${\mathbf A}=\sum _{i=1}^{n}\sum _{j=1}^{p} \mathbf {A}_{\textit {ij}}$, where **A**_*ij*_ is a *n*×*p* matrix with all zero elements except *a*_*ij*_ being at row *i* and column *j*. Let **B** be a *n*×*p* matrix consists of all *b*_*ij*_s. Then (9) is equivalent to

(10) $$ \begin{array} {ll} &{\mathop{\text{arg min}}\limits_{\mathbf{X}}}\left\{L(\mathbf{X}, \widetilde{\mathbf{W}}, \widetilde{\mathbf{Z}}, \widetilde{\mathbf{Y}})\right\}\\ &={\mathop{\text{arg min}}\limits_{\mathbf{X}}}\left\{\|\mathbf{A}\cdot(\mathbf{B}-\mathbf{ X})\|_{F}^{2}+2\alpha_{1}\|\mathbf{ X}\|_{*}\right\}\\ &={\mathop{\text{arg min}}\limits_{\mathbf{ X}}}\left\{\mathop{\sum}\limits_{i=1}^{n}\mathop{\sum}\limits_{j=1}^{p} \left(a_{ij}b_{ij}-<\mathbf{A}_{ij}, \mathbf{ X}>\right)^{2}\,+\,2\alpha_{1}\|\mathbf{ X}\|_{*}\right\}\\ &={\mathop{\text{arg min}}\limits_{\mathbf{ X}}}\left\{\|\mathbf{ C}-\mathbf{X}\|_{F}^{2} +2\alpha_{1}'\|\mathbf{ X}\|_{*}\right\}, \end{array}  $$

where $\mathbf {C}=(1+\rho)^{-1}\sum _{i=1}^{n}\sum _{j=1}^{p} a_{\textit {ij}}b_{\textit {ij}}\mathbf { A}_{\textit {ij}}=(1+\rho)^{-1}\mathbf {A}\cdot \mathbf { A}\cdot \mathbf { B}$ and *α*1′=*α*_1_/(1+*ρ*).

An additional pdf file shows more detailed derivation (see Section 3 in Additional file 1). Thus solving **X** reduces to a soft thresholding of singular values in the singular value decomposition of **C**. In particular, let {**u**_*i*_}, {**v**_*i*_} and {*σ*_*i*_} be the left singular vectors, the right singular vectors and the singular values of **C**, respectively. We can update **X** from

(11) $$\begin{array}{@{}rcl@{}} \mathbf{ X}=\sum_{i=1}^{r}(\sigma_{i}-\alpha_{1}')_{+}\mathbf{u}_{i}\mathbf{v}_{i}^{T}, \end{array} $$

where *r* is the rank of **C** and (*a*)_+_= max{*a*,0}.

If $(\widetilde {\mathbf {X}}, \widetilde {\mathbf {W}}, \widetilde {\mathbf {Y}})$ is obtained, solving **Z** from (7) becomes

(12) $$\begin{array}{@{}rcl@{}} \begin{array} {ll} &{\mathop{\text{arg\, min}}\limits_{\mathbf{Z}}}\left\{L(\widetilde{\mathbf{X}},\widetilde{\mathbf{W}}, {\mathbf{Z}}, \widetilde{\mathbf Y})\right\}\\ &= {\mathop{\text{arg\, min}}\limits_{\mathbf{Z}}}\left\{\frac{1}{2}\|\widetilde{\mathbf{X}}+\widetilde{\mathbf{ Y}}-{\mathbf Z}\|_{F}^{2} + \frac{\alpha_{2}}{\rho}\mathop{\sum}\limits_{i=1}^{n}\|{\mathbf z}_{i}\|_{\text{TV}}\right\}\\ &={\mathop{\text{arg\, min}}\limits_{{\mathbf Z}}}\left\{\mathop{\sum}\limits_{i=1}^{n} \left[ \frac{1}{2} \|\widetilde{\mathbf{x}}_{i}+\widetilde{\mathbf y}_{i}-{\mathbf z}_{i}\|_{2}^{2}+\frac{\alpha_{2}}{\rho}\|{\mathbf z}_{i}\|_{\text{TV}}\right]\right\}. \end{array} \end{array} $$

Thus for each 1≤*i*≤*n*, we can update **z**_*i*_ using the fused lasso signal approximation algorithm in [49].

We summarize the above iterations in the following Algorithm 1.



The objective function in (3) is a bi-convexity optimization problem. Two matrices **W** and **X** are alternatingly updated with the other held fixed until reaching convergence. However, the initial points **X** ^(0)^ and **W** ^(0)^ may affect the final solution. Rousseeuw and Driessen [50] suggests a multi-start iterative strategy in general. In our applications, we found initial values generated from both RPLA and CPLA work very well.

### Tuning parameter selection

Tuning parameter selection is always challenging in penalized optimization. It is computationally expensive to extensively search an optimal (*α*_1_,*α*_2_,*β*) in (7). Here we propose some strategies on selecting three types tuning parameters. This strategy works surprisingly well during the implementation of both synthetic data and two real data analysis.

1) Type 1 tuning parameters including *α*_1_ and *α*_2_ control the hidden recurrent copy number structure. Between them, *α*_1_ is the most important one. We follow the discussions in [41] and let $\alpha _{1}=(\sqrt {n}+\sqrt {p})\widehat {\sigma }$, where $\widehat {\sigma }=1.48MAD$, where *M* *A* *D*=median{|**D**−median(**D**)|}. After *α*_1_ is determined, we fix *α*_2_=0.1*α*_1_.

2) Type 2 tuning parameter includes *β*, controlling the ratio of individual CNVs (outliers). Refer to (Gao X, Fang Y: Penalized weighted least squares for outlier detection and robust regression, Under revision), we provide a Bayesian understanding of the weight parameter penalty term in a multivariate regression framework, where the robust estimation of the coefficients vector is a posterior mode if *ν*=1/*w* when *ν* has a Type I Pareto prior distribution *π*(*ν*)∝*ν* ^1−*β*^ *I*(*ν*≥1), where *β*_0_=1 is the uniform prior and *β*_0_=2 is the Jeffrey’s prior. This motivates us to select the tuning parameter $\beta =\widehat {\sigma }^{2}\beta _{0}$ with *β*_0_=1 or 2, where $\widehat {\sigma }$ is a robust measurement of the noises’ variability. For example, we let $\widehat {\sigma }=1.4826 MAD$ in real implementation [51]. An additional pdf file shows more details on this Bayesian understanding (see Section 2 in Additional file 1).

3) Type 3 tuning parameter *ρ* is the parameter controls the convergence of the algorithm. We let *ρ*=0.1*σ*_**D**_, where *σ*_**D**_ is the maximum singular value of matrix **D**, and adaptively tuned during the iteration following [43]. On the one hand, if the primal residual (the maximal singular value of **X**−**Z**) is 10 times of the dual residual (the maximal singular value of $\rho ({\mathbf Z}-\widetilde {\mathbf Z})$), then *ρ* is doubled. On the other hand, if the dual residual is 10 times of the primal residual, then *ρ* is halved. We keep updating *ρ* during the iteration steps in Algorithm 1 until converge.

## Results and discussion

### Results

#### Synthetic data sets

We generate multiple synthetic data sets with 50 samples and 300 probes from

(13) $$\begin{array}{@{}rcl@{}} {\mathbf D}={\mathbf X}+{\mathbf E}+{\boldsymbol{\varepsilon}}. \end{array} $$

This means that the synthetic data is consistent with the mean shift model studied in RPLA, and our WPLA model is actually misspecified under (13). However, we will show that WPLA still performs the best among all these three methods in all numerical experiments.

We consider twelve different combinations of six recurrent CNV scenarios (**X**) discussed in [31] and two types of random errors (***ε***). All individual sparse signals (**E**) are generated similar to [41]. We summarize all details as follows.

- (Recurrent CNVs: **X**) Six different types recurrent CNV regions are listed in Table 1. Here all gains (+) and losses (-) have the true signal value of 1 and -1, respectively.

**Table 1:** 12 Synthetic data settings: “+” means gains, “-” means losses

	Case 1 & 7				Case 2 & 8			
Sample	[76, 85]	[86, 95]	[96, 105]	[106, 125]	[76, 85]	[86, 95]	[96, 105]	[106, 125]
1-10	+	+					-	-
11-20	+	+					-	-
21-30	+	+			+	+		
31-40	+	+			+	+		
41-50	+	+			+	+		
	Case 3 & 9				Case 4 & 10			
Sample	[76, 85]	[86, 95]	[96, 105]	[106, 125]	[76, 85]	[86, 95]	[96, 105]	[106, 125]
1-10	-	-			+	+	+	
11-20	-	-			+	+	+	
21-30	+	+				+	+	
31-40	+	+					-	-
41-50	+	+					-	-
	Case 5 & 11				Case 6 & 12			
Sample	[76, 85]	[86, 95]	[96, 105]	[106, 125]	[76, 85]	[86, 95]	[96, 105]	[106, 125]
1-10	+	+	+		+	+	+	
11-20					-	-	-	
21-30	-	-	-					
31-40	+	+	+				+	+
41-50	+	+	+				-	-
- (Individual CNVs: **E**) Each sample includes an individual CNV region with a length of 20 probes. This region is randomly located outside of recurrent regions, with intensities randomly selected from {-2, -1, 1, 2}.
- (Random error: ***ε***) Two types of noises are considered for all probes: 1) Case 1-6 have Gaussian noises with *σ*=0.3; 2) Case 7-12 have contaminated noises from 0.5*t*(1), where *t*(1) indicates a *t* distribution with degrees of freedom of 1. We take *t*(1) distribution as a heavily contaminated example since t-distribution is often used for quantifying the thicker tails of genetic data as mentioned in [52, 53]. In addition, no finite variance for *t*(1). Thus Case 7-12 are corresponding heavily contaminated scenarios parallel to Case 1-6.

**Fig. 1:**
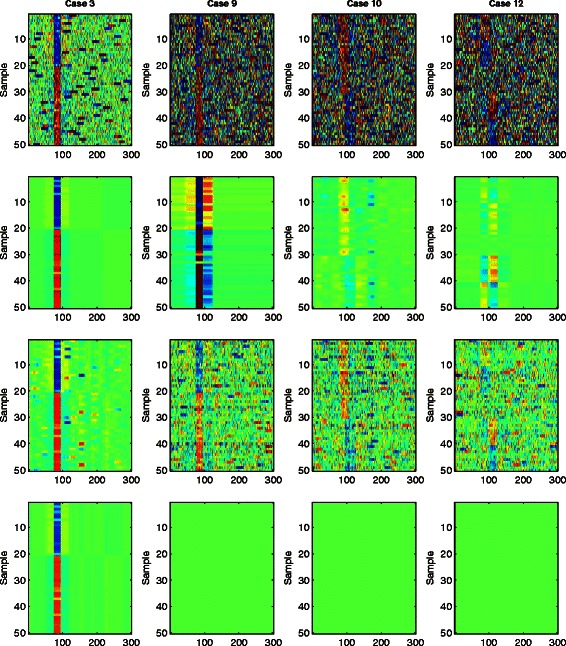
Sample synthetic data and its low-rank output. Column 1: Case 3; Column 2: Case 9; Column 3: Case 10; Column 4: Case 12. Row 1: Input observations; Row 2: WPLA recovery; Row 3: RPLA recovery; Row 4: CPLA recovery

In Fig. 1, we provide some heat maps of 4 synthetic data sets for Case 3 (Column 1), Case 9 (Column 2), Case 10 (Column 3), and Case 12 (Column 4). The input data matrix is plotted in Row 1, with corresponding recurrent CNV regions recovery from WPLA, RPLA and CPLA plotted in Row 2, Row 3, and Row 4, respectively. Under normal noises with individual-specific CNVs in Case 3, both WPLA and CPLA provide almost perfect mappings of the true hidden low-rank matrix **X**. Although RPLA provides a slightly more noisy output than WPLA and CPLA, it can still recover the recurrent CNV region reasonably well. However, under a parallel heavily contaminated case in Case 9, both CPLA and RPLA lose their abilities of detecting recurrent CNVs. On the one hand, CPLA provides a zero estimation for **X** and treat the entire data as random noises. On the other hand, RPLA provides a considerably noisy estimation of **X**. Compared with both RPLA and CPLA, WPLA provides a much more efficient recovery of those recurrent CNV regions, although there may have some false positives around those underlying CNV regions (See all plots at column 2 from Fig. 1). Similarly, WPLA shows dramatic improvement from RPLA and CPLA in detecting recurrent CNVs under Case 10 and 12, where the underlying recurrent CNV regions are much more complicated.

**Fig. 2:**
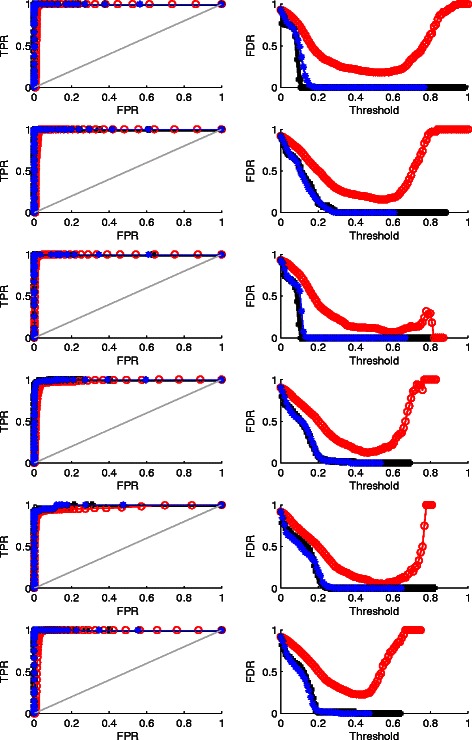
Recurrent CNVs Detection Result for Case 1-6, where random noise is Gaussian. Row 1-6 are for Case 1-6, respectively. Column 1: ROC curves (the higher, the better); Column 2: FDR curves (the lower, the better). Black: WPLA output; Red: RPLA output; Blue: CPLA output

**Fig. 3:**
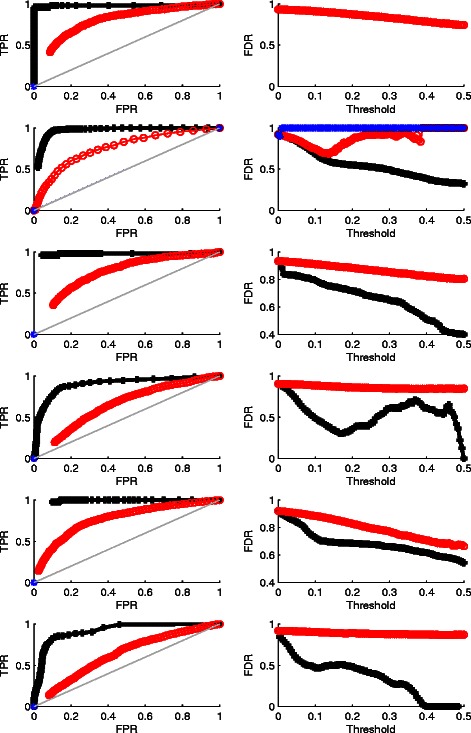
Recurrent CNVs Detection Result for Case 7-12, where data is heavily contaminated. Row 1-6 are for Case 7-12, respectively. Column 1: ROC curves (the higher, the better); Column 2: FDR curves (the lower, the better). Black: WPLA output; Red: RPLA output; Blue: CPLA output. Some of CPLA curves disappear since CPLA does not produce any recurrent CNVs region when data is heavily contaminated

To further evaluate the performance of WPLA in terms of recurrent CNV detection, we compute both the true positive rate (TPR) and the false positive rate (FPR), and estimate the false discovery rate (the false positive number out of total number of recurrent CNVs detected). In particular, we report both the receiver operation characteristic (ROC) curves (TPR vs FPR) and the false discovery rate (FDR=FP/(TP+FP)) based upon a sequence of cutoff values for CNVs. ROC and FDR curves from Case 1-6 and Case 7-12 are plotted and compared in Figs. 2 and 3, respectively. In each figure, all left panels are ROC curves (the higher, the better), all right panels are FDR curves (the lower, the better).

**Fig. 4:**
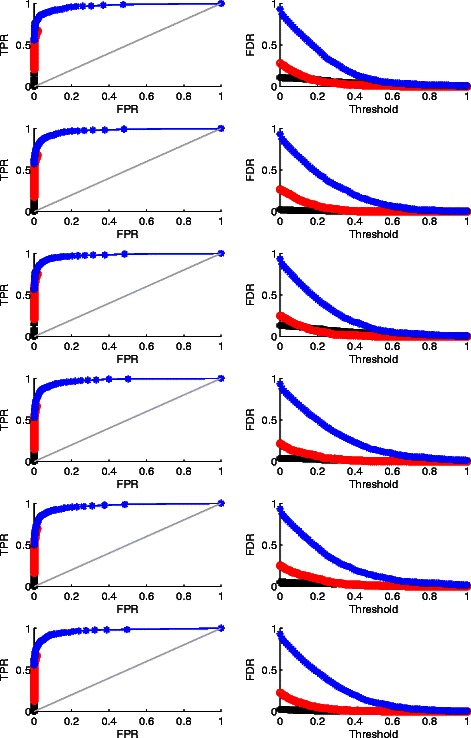
Individual CNVs Detection Result for Case 1-3. Row 1-6 are for Case 1-6, respectively. Column 1: ROC curves (the higher, the better); Column 2: FDR curves (the lower, the better). Black: WPLA output; Red: RPLA output; Blue: CPLA output

We also plot both ROC and FDR curves regarding the individual-specific CNVs detection for Case 1-6 in Fig. 4. The comparison between WPLA and RPLA or CPLA under other cases exhibit the similar patterns and are omitted due to the space limit.

We have the following findings. 1) When the random noise is Gaussian, all three methods perform well in terms of the recurrent CNVs detection; there is still some advantages of WPLA and CPLA over RPLA by producing smaller false discovery rate. 2) Among all three methods, CPLA performs the worst in terms of individual CNVs detection. This is not surprising since CPLA is only designed for robust detection of recurrent CNVs, not for individual CNVs. 3) When the data is heavily contaminated in addition to the existence of individual CNVs, WPLA beats both two other methods considerablyby producing much higher ROC curves and much lower FDR curves. In this situation, CPLA loses control of detecting recurrent CNVs completely and treat the input data as noise most of the time.Therefore, some of CPLA curves disappear in Fig. 3 since CPLA does not produce any recurrent CNVs region when data is heavily contaminated. Overall, WPLA has strong robust properties when data is heavily contaminated; it is as efficient as existing robust methods when the random noise is normally distributed. In addition, WPLA has the ability of simultaneous detection of both recurrent and individual CNVs.

#### Real applications

We apply WPLA to three independent real data sets: chromosome 17 from Pollack data [54], chromosome 17 from Bekhouche data [55], and chromosome 11 from Wang data [16]. The first two data sets were also analyzed by [41]. The Pollack data consists of log2 intensities at 382 probes from 44 breast tumors samples, while the Bekhouche data is a much larger data set including 7727 probes from 173 breast tumors. Compared with the other two data sets, Wang data is the newest with the highest resolution, and has much smaller sample size with only 12 pig samples: one Asian wild boar population, six Chinese indigenous breeds, and two European commercial breeds. Due to the small sample size and high resolution, we only analyze one segment of chromosome 11 (between 62,001,001 and 71,997,810), where three CNVs regions were confirmed by the quantitative real-time PCR analysis. Thus, Wang data consists of 3766 probes from 12 samples.

**Fig. 5:**
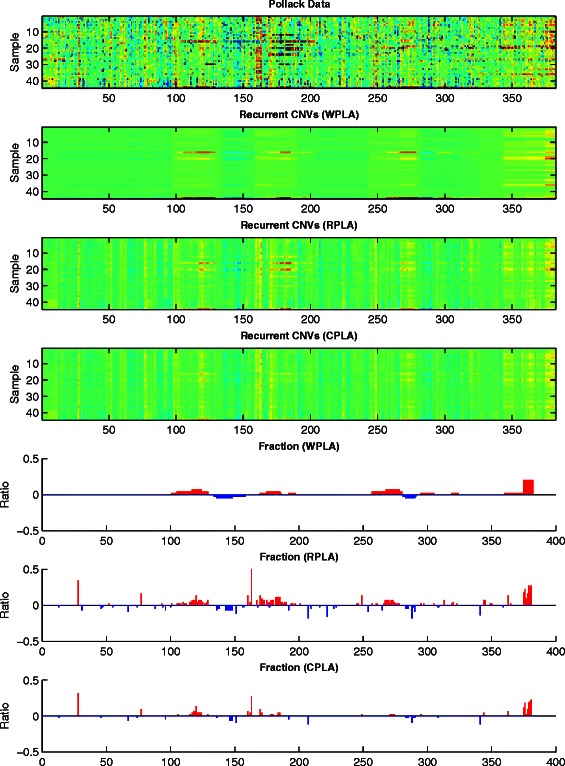
Pollack data analysis results. Row 1: Input observation matrix; Row 2: recurrent CNVs low-rank matrix recovery from WPLA; Row 3: recurrent CNVs low-rank matrix recovery from RPLA; Row 4: recurrent CNVs low-rank matrix recovery from CPLA; Row 5: recurrent CNVs frequency output from WPLA; Row 6: recurrent CNVs frequency output from RPLA; Row 7: recurrent CNVs frequency output from CPLA

**Fig. 6:**
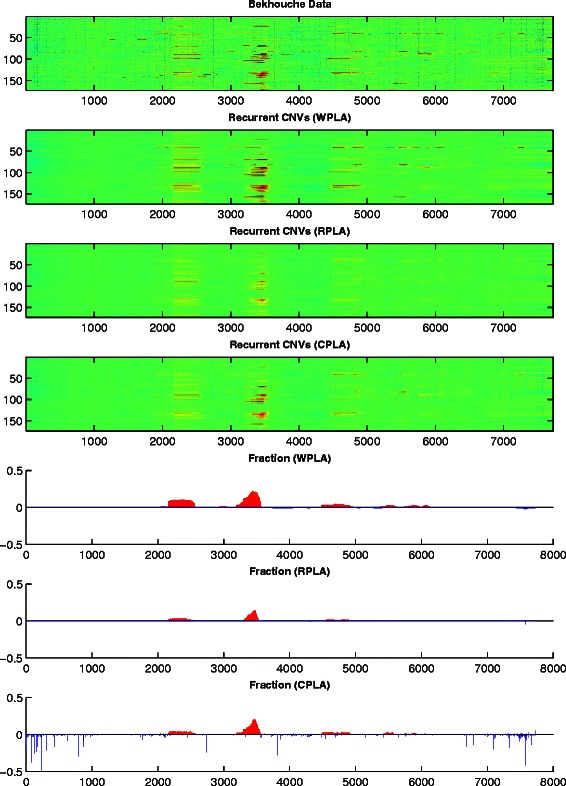
Bekhouche data analysis results. Row 1: Input observation matrix; Row 2: recurrent CNVs low-rank matrix recovery from WPLA; Row 3: recurrent CNVs low-rank matrix recovery from RPLA; Row 4: recurrent CNVs low-rank matrix recovery from CPLA; Row 5: recurrent CNVs frequency output from WPLA; Row 6: recurrent CNVs frequency output from RPLA; Row 7: recurrent CNVs frequency output from CPLA

**Fig. 7:**
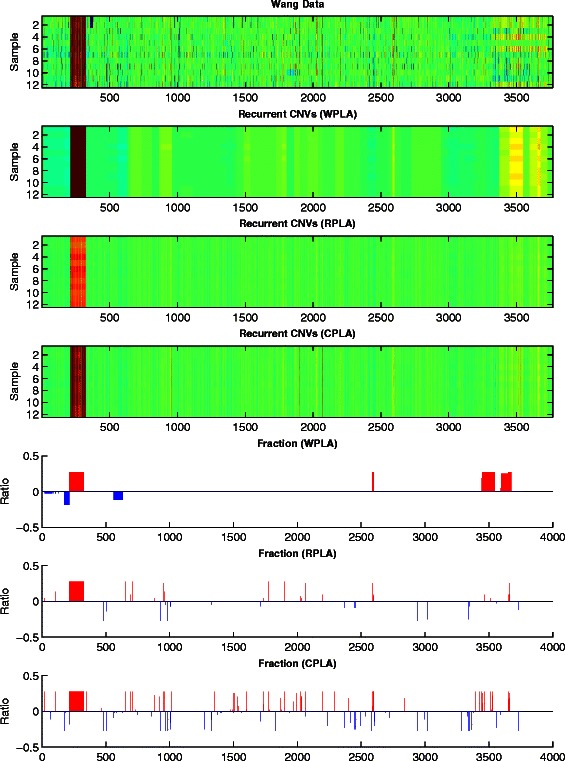
Wang data analysis results. Row 1: Input observation matrix; Row 2: recurrent CNVs low-rank matrix recovery from WPLA; Row 3: recurrent CNVs low-rank matrix recovery from RPLA; Row 4: recurrent CNVs low-rank matrix recovery from CPLA; Row 5: recurrent CNVs frequency output from WPLA; Row 6: recurrent CNVs frequency output from RPLA; Row 7: recurrent CNVs frequency output from CPLA

Figures 5, 6 and 7 give the heatmaps of the Pollack data, the Bekhouche data, and the Wang data and their corresponding recurrent CNVs analysis results, respectively. In each figure, we plot the original data in Row 1 and report the detected recurrent CNVs regions from WPLA, RPLA, CPLA in Row 2-4. Corresponding gains and losses ratios out of all samples profiles are also reported in Row 5-7 in each figure, where a CNV is claimed if the estimation $|\widehat x_{\textit {ij}}|>0.225$.

For the Pollack data and Wang data, three methods produce more discrepant CNV regions, where results from WPLA are smoother than two other methods. For the Bekhouche data, all three methods produce more consistent recurrent CNV regions. Overall, among all detected recurrent CNV regions, WPLA turns to produce higher frequency ratio among all samples, which is reasonable for recurrent CNVs detection. For example, WPLA detects the recurrent CNVs region where gene ERBB2 and C17orf37 are located (at around probe 3460). This result is consistent with scientific discoveries since both of those two genes are claimed to be related breast cancer [41, 56]. It is worthwhile to point it out that WPLA detects this recurrent region at a much higher frequency ratio than both RPLA and CPLA.

### Discussion

All numerical experiments have been done under fixed tuning parameters. There is still some potential improvement if all parameters are optimally tuned. However, the computation must be much more expensive. It is worthwhile to investigate some more effective ways of tuning parameter selection methods in future studies.

## Conclusions

In this paper, we propose a novel robust method for recurrent copy number variation detection. This method is unique by assigning a weight parameter to each probe of every sample. Thus, all the individual effects are related to a weight matrix **W**, which is estimated data adaptively, together with the low-rank approximation. As a result, a robust detection of recurrent CNVs is obtained by shrinking all weights from some small values (because of individual-specific effects) to 1 (no individual effects). This proposed method has two important results: efficient detection of both recurrent CNVs and individual-specific CNVs, strong robustness in dealing with severe data contamination.

We have applied the proposed method to three real data sets and twelve synthetic data sets generated from six different types of recurrent CNVs associated with either normal random errors or heavily contaminated errors. The numerical experiment has demonstrated its superior performance of recovering recurrent CNV patterns from raw data under different scenarios.Compared with two other recent methods, it has the best ability of simultaneous detection of both recurrent and individual-specific CNVs under normal random errors. More importantly, the proposed method is the only one which can effectively recover the recurrent CNVs region when the data is heavily contaminated.

## Additional file

Additional file 1 **Supplementary Material.** Supplementary Material is also available online under the name of “wccna_suppl2.pdf” and PDF format. This Supplementary Material provides additional proofs and some more mathematical details associated with the proposed method. In particular, there are three sections in the Supplementary Material. Section 1 is on the link between WPLA and a redescending M-estimation; Section 2 is on Bayesian understanding of WPLA; Section 3 is on more detailed derivation of some equations in Algorithm 1. (PDF 185 kb)
