# Supplementary material for: Penalized weighted low-rank approximation for robust recovery of recurrent copy number variations
Source: BMC Bioinformatics. 2015 Dec 10;16:407. doi: 10.1186/s12859-015-0835-2 (PMC4676147; doi:10.1186/s12859-015-0835-2)
Supplement: Supplementary file 1 — Supplementary Material. Supplementary Material is also available online under the name of “wccna_suppl2.pdf” and PDF format. This Supplementary Material provides additional proofs and some more mathematical details associated with the proposed method. In particular, there are three sections in the Supplementary Material. Section 1 is on the link between WPLA and a redescending M-estimation; Section 2 is on Bayesian understanding of WPLA; Section 3 is on more detailed derivation of some equations in Algorithm 1. (PDF 185 kb) [file 12859_2015_835_MOESM1_ESM.pdf]

Supplementary Material
Penalized Weighted Low-rank Approximation for Robust Recovery of Recurrent Copy Number Variations

Xiaoli Gao
Department of Mathematics and Statistics
University of North Carolina at Greensboro

1 Link between WPLA and a redescending M-estimation

For simplicity, we only discuss the association of WPLA and a redescending M-estimation under a special case, where $x_{i1} = \cdots = x_{ip}$ for $1 \le i \le n$. We denote x_i the unique element in row i.

Consider a penalized weight estimation with a concomitant scale,

$$(\widehat{\mathbf{X}}, \widehat{\mathbf{W}}, \widehat{\sigma}) = \arg\min \left\{ \frac{1}{2\sigma^2} \sum_{i=1}^{n} \sum_{j=1}^{p} w_{ij}^2 (d_{ij} - x_i)^2 + \sum_{i=1}^{n} \sum_{j=1}^{p} \beta |\log(w_{ij})| + cnp \log(\sigma) \right\}. \quad (1.1)$$

Similarly, we define a robust M-estimation,

$$(\widehat{\mathbf{X}}_M, \widehat{\sigma}_M) = \arg\min \left\{ \sum_{i=1}^{n} \sum_{j=1}^{p} \rho_\beta \left(\frac{d_{ij} - x_i}{\sigma} \right) + 2cnp \log(\sigma) \right\}, \quad (1.2)$$

where

$$\rho_\beta(t) = \begin{cases} \beta \log(t^2/\beta) + \beta & \text{if } |t| > \sqrt{\beta}, \\ t^2 & \text{if } |t| \le \sqrt{\beta}. \end{cases} \quad (1.3)$$

Let $\psi_\beta(t) = \rho'_\beta(t)$, its derivative. Then

$$\psi_\beta(t) = \begin{cases} 2\beta/t, & \text{if } |t| > \sqrt{\beta}, \\ 2t, & \text{if } |t| \le \sqrt{\beta}, \end{cases} \quad (1.4)$$

We have the following result.

Theorem 1 *For any given β and c, the robust M estimation $\widehat{\mathbf{X}}_M$ from* (1.2) *is the same as the penalized weight estimation $\widehat{\mathbf{X}}$ from* (1.1).

Proof.

We first consider joint KKT equations under (1.2),

$$\begin{cases} \sum_{i=1}^n \sum_{j=1}^p \psi_\beta \left(\dfrac{d_{ij} - x_i}{\sigma} \right) = 0 \\ \dfrac{\partial}{\partial \sigma} \left(\sum_{i=1}^n \sum_{j=1}^p \rho_\beta \left(\dfrac{d_{ij} - x_i}{\sigma} \right) + 2cnp \log \sigma \right) = 0 \end{cases}$$

We can check

$$\frac{\partial}{\partial \sigma}(\rho_\beta(t/\sigma) + 2cnp\log(\sigma)) = \begin{cases} -2\beta/\sigma + 2cnp/\sigma & \text{if } |t| > \sqrt{\beta}\sigma \\ -2t^2/\sigma^3 + 2cnp/\sigma & \text{if } |t| \le \sqrt{\beta}\sigma \end{cases} \tag{1.5}$$

Replace t by $d_{ij} - x_i$ and set (1.2) to be 0, we have

$$cnp - \sum_{(i,j)\in\widehat{O}} \beta - \sum_{(i,j)\in\widehat{G}} (d_{ij} - x_i)^2/\sigma^2 = 0,$$

where $\widehat{O} = \{(i,j) : |d_{ij} - x_i| > \sqrt{\beta}\sigma\}$ and $\widehat{G} = \{(i,j) : |d_{ij} - x_i| \le \sqrt{\beta}\sigma\}$. Denote $r_{ij} = d_{ij} - x_i$. Then we have

$$\widehat{\sigma}^2 = \sum_{(i,j)\in\widehat{G}} \widehat{r}_{ij}^2/(cnp - \beta|\widehat{O}|), \tag{1.6}$$

where $|\widehat{O}|$ is the cardinal value of set $\widehat{O}$. We now consider joint KKT of the penalized objective function in (1.1). From the derivative on $\mathbf{w}$, we obtain

$$w_{ij} = \begin{cases} \sqrt{\beta}(\sigma/|\widehat{r}_{ij}|) & \text{if } |\widehat{r}_{ij}| > \sigma\sqrt{\beta} \\ 1 & \text{if } |\widehat{r}_{ij}| \le \sigma\sqrt{\beta} \end{cases} \tag{1.7}$$

From the derivative on σ,

$$cnp\sigma^2 = \sum_{i=1}^n \sum_{j=1}^p w_{ij}^2 (d_{ij} - x_i)^2 = \sum_{(i,j)\in\widehat{G}} \widehat{r}_{ij}^2 + \sum_{(i,j)\in\widehat{O}} (\widehat{w}_{ij}^2 \widehat{r}_{ij}^2). \tag{1.8}$$

Combining with (1.8) and (1.7), we can also obtain (1.6).

Finally, plugging in (1.7) in (1.1), we are able to obtain the concomitant M-estimation ρ function in (1.2). □

2 A Bayesian understanding of WPLA

Consider a multivariate regression model $d_{ij} = x_{ij} + \varepsilon_{ij}$, where $\varepsilon_{ij} \sim N(0, \sigma_{ij}^2)$ with $\sigma_{ij}^2 = \sigma^2/w_{ij}^2$. Without loss of generality, we assume $\sigma^2 = 1$. We propose a Bayesian model based on which the posterior distribution of $\nu_{ij} = 1/w'_{ij}$. Denote $\boldsymbol{\nu}$ the $n \times p$ matrix consisting of ν_{ij}s. Let $\pi(x_{ij})$ and $\pi(\nu_{ij})$ be the independent prior distributions of x_{ij} and ν_{ij}, respectively. Assume non-informative priors $\pi(x_{ij}) \propto 1$. Also assume that the prior distribution of ν_{ij} is Type I Pareto distribution with hyper-parameter $\beta \geq 1$; that is,

$$\pi(\nu_{ij}) \propto \nu_{ij}^{1-\beta} I(\nu_i \geq 1), \quad \text{for } 1 \leq i \leq n, 1 \leq j \leq p, \tag{2.1}$$

where $I(\cdot)$ is the indicator function. In particular, it is the uniform non-informative prior when $\beta = 1$ and it is Jeffreys non-informative prior when $\beta_0 = 2$.

Then the joint posterior distribution of the parameters is equal to

$$\pi(\mathbf{X}, \boldsymbol{\nu}|\mathbf{y}) \propto \prod_{i=1}^{n}\prod_{j=1}^{p} \nu_{ij}^{-\beta} \exp\left\{-\frac{1}{2}\sum_{i=1}^{n}\sum_{j=1}^{p} \frac{1}{\nu_{ij}^2}(d_{ij} - x_{ij})^2\right\}. \tag{2.2}$$

Then the mode, $(\widehat{\mathbf{X}}, \widehat{\boldsymbol{\nu}})$ of the above posterior distribution is

$$(\widehat{\mathbf{X}}, \widehat{\boldsymbol{\nu}}) \quad = \quad \arg\min\left\{\sum_{i=1}^{n}\sum_{j=1}^{p} \frac{1}{\nu_{ij}^2}(d_{ij} - x_{ij})^2 + \sum_{i=1}^{n}\sum_{j=1}^{p} 2\beta|\log(\nu_{ij})|\right\}. \tag{2.3}$$

Replacing $1/\nu_{ij}$ by w_{ij}, (2.3) is equivalent to

$$(\widehat{\mathbf{X}}, \widehat{\mathbf{W}}) \quad = \quad \arg\min\left\{\frac{1}{2}\sum_{i=1}^{n}\sum_{j=1}^{p} w_{ij}^2(d_{ij} - x_{ij})^2 + \sum_{i=1}^{n}\sum_{j=1}^{p} \beta|\log(w_{ij})|\right\}. \tag{2.4}$$

If $\sigma \neq 1$, and let $\pi(\sigma^2) \propto 1/\sigma^2$ be a prior distribution of σ^2, then (2.4) becomes

$$(\widehat{\mathbf{X}}, \widehat{\mathbf{W}}) = \arg\min\left\{\frac{1}{2}\sum_{i=1}^{n}\sum_{j=1}^{p} w_{ij}^2(d_{ij} - x_{ij})^2 + \sum_{i=1}^{n}\sum_{j=1}^{p} \beta\widehat{\sigma}^2|\log(w_{ij})|\right\}$$

and

$$\widehat{\sigma}^2 = \frac{1}{np+2}\sum_{i=1}^{n}\sum_{j=1}^{p} \widehat{w}_{ij}^2(d_{ij} - x_{ij})^2.$$

3 Derivation of some equations in Algorithm 1

First, equation (7) is a dual formulation of equation (3). To obtain equation (8), we take derivative of (7), which reduces to the derivative on

$$L(\widetilde{\mathbf{X}}, \mathbf{W}) = \frac{1}{2}\sum_{i=1}^{n}\sum_{j=1}^{p} w_{ij}^2 (d_{ij} - \widetilde{x}_{ij})^2 + \beta \sum_{i=1}^{n}\sum_{j=1}^{p} |\log(w_{ij})|$$

for $w_{ij} < 1$. By simple algebra computation, we obtain $w_{ij} = \dfrac{\sqrt{\beta}}{|d_{ij} - \widetilde{x}_{ij}|}$ for $w_{ij} < 1$, that is $|d_{ij} - \widetilde{x}_{ij}| > \sqrt{\beta}$. Therefore, $w_{ij} = 1$ for $|d_{ij} - \widetilde{x}_{ij}| \leq \sqrt{\beta}$.

We now provide a detailed derivation of (9). Once $(\widetilde{\mathbf{W}}, \widetilde{\mathbf{Z}}, \widetilde{\mathbf{Y}})$ is obtained, solving $\mathbf{X}$ is equivalent to minimizing

$$\begin{aligned} L(\mathbf{X}, \widetilde{\mathbf{W}}, \widetilde{\mathbf{Z}}, \widetilde{\mathbf{Y}}) &= \frac{1}{2}\|\widetilde{\mathbf{W}} \cdot (\mathbf{D} - \mathbf{X})\|_F^2 + \alpha_1 \|\mathbf{X}\|_* + < \widetilde{\mathbf{Y}}, \mathbf{X} - \widetilde{\mathbf{Z}} > + (\rho/2)\|\mathbf{X} - \widetilde{\mathbf{Z}}\|_F^2 \\ &= \frac{1}{2}\mathrm{Tr}((\widetilde{\mathbf{W}} \cdot \mathbf{D})'(\widetilde{\mathbf{W}} \cdot \mathbf{D})) - \mathrm{Tr}((\widetilde{\mathbf{W}} \cdot \mathbf{D})'(\widetilde{\mathbf{W}} \cdot \mathbf{X})) + \frac{1}{2}\mathrm{Tr}((\widetilde{\mathbf{W}} \cdot \mathbf{X})'(\widetilde{\mathbf{W}} \cdot \mathbf{X})) \\ &\quad + \mathrm{Tr}(\widetilde{\mathbf{Y}}'\mathbf{X}) - \mathrm{Tr}(\widetilde{\mathbf{Y}}'\widetilde{\mathbf{Z}}) + \frac{\rho}{2}\mathrm{Tr}(\mathbf{X}'\mathbf{X}) + \frac{\rho}{2}\mathrm{Tr}(\widetilde{\mathbf{Z}}'\widetilde{\mathbf{Z}}) - \rho\mathrm{Tr}(\widetilde{\mathbf{Z}}'\mathbf{X}) + \alpha_1\|\mathbf{X}\|_* \end{aligned}$$

Thus, minimizing $L(\mathbf{X}, \widetilde{\mathbf{W}}, \widetilde{\mathbf{Z}}, \widetilde{\mathbf{Y}})$ is equivalent to minimizing

$$\frac{1}{2}\mathrm{Tr}((\widetilde{\mathbf{W}} \cdot \mathbf{X})'(\widetilde{\mathbf{W}} \cdot \mathbf{X})) + \frac{\rho}{2}\mathrm{Tr}(\mathbf{X}'\mathbf{X}) + \mathrm{Tr}((\widetilde{\mathbf{Y}}' - \rho\widetilde{\mathbf{Z}}')\mathbf{X}) - \mathrm{Tr}((\widetilde{\mathbf{W}} \cdot \mathbf{D})'\widetilde{\mathbf{W}} \cdot \mathbf{X}) + \alpha_1\|\mathbf{X}\|_*.$$

That is,

$$\frac{1}{2}\sum_{i=1}^{n}\sum_{j=1}^{p} \widetilde{w}_{ij}^2 x_{ij}^2 + \frac{\rho}{2}\sum_{i=1}^{n}\sum_{j=1}^{p} x_{ij}^2 + \sum_{i=1}^{n}\sum_{j=1}^{p} (\widetilde{y}_{ij} - \rho\widetilde{z}_{ij} - \widetilde{w}_{ij}^2 d_{ij}) x_{ij} + \alpha_1\|\mathbf{X}\|_*.$$

Writing this into a quadratic format, minimizing $L(\mathbf{X}, \widetilde{\mathbf{W}}, \widetilde{\mathbf{Z}}, \widetilde{\mathbf{Y}})$ is equivalent to minimizing

$$\sum_{i=1}^{n}\sum_{j=1}^{p} (\widetilde{w}_{ij}^2 + \rho)\left[x_{ij} - \frac{\widetilde{w}_{ij}^2 d_{ij} + \rho\widetilde{z}_{ij} - \widetilde{y}_{ij}}{\widetilde{w}_{ij}^2 + \rho}\right]^2 + 2\alpha_1\|\mathbf{X}\|_*$$

Here we give more details on the last "=" of Equation (10). Denote $\|\mathbf{X}\|_{L_2(\Pi)}^2 = \sum_{i,j} < \mathbf{X}, \mathbf{A}_{ij} >$.

Then

$$\begin{aligned} &= \arg\min_{\mathbf{X}} \left\{ \sum_{i=1}^{n}\sum_{j=1}^{p} (a_{ij}b_{ij} - < \mathbf{A}_{ij}, \mathbf{X} >)^2 + 2\alpha_1\|\mathbf{X}\|_* \right\} \\ &= \arg\min_{\mathbf{X}} \left\{ \|\mathbf{X}\|_{L_2(\Pi)}^2 - 2\sum_{i=1}^{n}\sum_{j=1}^{p} a_{ij}b_{ij}\mathbf{A}_{ij}, \mathbf{X} > + 2\alpha_1\|\mathbf{X}\|_* \right\} \\ &= \arg\min_{\mathbf{X}} \left\{ (1+\rho)\|\mathbf{X}\|_2^2 - 2\sum_{i=1}^{n}\sum_{j=1}^{p} a_{ij}b_{ij}\mathbf{A}_{ij}, \mathbf{X} > + 2\alpha_1\|\mathbf{X}\|_* \right\} \\ &= \arg\min_{\mathbf{X}} \left\{ (1+\rho)^{-1}\|\mathbf{A} \cdot \mathbf{A} \cdot \mathbf{B} - \mathbf{X}\|_F^2 + 2\alpha_1/(1+\rho)\|\mathbf{X}\|_* \right\}, \end{aligned}$$

where the last "=" are from (3.1) in [1].

References

[1] V. Koltchinskii, K. Lounici, and A. Tsybakov. Nuclear norm penalization and optimal rates for noisy low rank matrix completion. *The Annals of Statistics*, 39:2302–2329, 2011.
